# Design considerations in the development of a core outcome set

**DOI:** 10.1186/1745-6215-14-S1-O68

**Published:** 2013-11-29

**Authors:** Nicola Harman, Iain Bruce, Peter Callery, Stephanie Tierney, Kevin O'Brien, Paula Williamson

**Affiliations:** 1 The Healing Foundation Cleft and Craniofacial Clinical Research Centre, University of Manchester, Manchester, UK; 2 Central Manchester University Hospitals NHS Foundation Trust, Manchester, UK; 3 School of Nursing, Midwifery & Social Work, University of Manchester, Manchester, UK; 4 Department of Biostatistics, University of Liverpool, Liverpool, UK

## 

No gold standard method currently exists for the development of a core outcome set (COS) although key issues to consider have been identified [1]. Methodological decisions in the design of a project to develop a COS for otitis media with effusion (OME) in children with cleft palate (CP) will be explained.

## Stakeholder groups

Clinicians involved in the management of OME were identified as: ENT surgeons; audiologists; cleft surgeons; speech and language therapists; specialist nurses. Discussions with CLAPA patient groups resulted in a Delphi for patients (7-11, 12-16 years), their parents and adults with CP and OME.

## Separate Delphi panels

A Delphi exercise has been undertaken with each clinical stakeholder group separately. This method does not assume that different stakeholder groups will have the same outcome priorities.

## Describing outcomes for patients and their parents

A lay description written for each outcome was reviewed by CLAPA groups and reading age assessed. Understanding was tested in a subsample using the think aloud method.

## Delivery of the Delphi online

Each round of the Delphi was delivered online using a bespoke system that managed participation invitations , reminders, data collection and reporting.

## Integration of clinical and patient views

Results of the separate Delphi surveys for each stakeholder group will be shared with all participants with an opportunity to re-score. Clinicians, adults, patients and their parents who have taken part in a Delphi panel/qualitative interview will be invited to a consensus meeting, facilitated by a Study Advisory Group and CLAPA members, to discuss the final COS.

## References

[B1] Williamson PR Altman DG Blazeby JM Clarke M Devane D Gargon E Tugwell P Developing core outcome sets for clinical trials: issues to consider Trials 2012 14 132 10.1186/1745-6215-13-132 22867278 PMC3472231

